# LAG-3 and 4-1BB identify dysfunctional antigen-specific T cells in the tumor microenvironment and combinatorial LAG-3/4-1BB targeting gives synergistic tumor control

**DOI:** 10.1186/2051-1426-3-S2-P328

**Published:** 2015-11-04

**Authors:** Jason B Williams, Brendan Horton, Yan Zheng, Thomas F Gajewski

**Affiliations:** 1 University of Chicago, Chicago, IL, USA; 2 University Hospitals Case Medical Center, Cleveland, OH, USA

## 

Although the presence of tumor-infiltrating lymphocytes (TILs) indicates an endogenous anti-tumor response, immune regulatory pathways can subvert the effector phase and enable tumor escape. One possible negative regulatory pathway is T cell-intrinsic anergy. Recently, we have shown that the transcription factor Egr2 is critical in controlling the anergic state using an in vitro model system. Gene expression profiling and Egr2 ChIP-Seq analysis revealed multiple Egr2-driven cell surface proteins in T cell anergy, including the inhibitory receptor LAG-3, but also the costimulatory receptor 4-1BB. We examined whether these surface proteins identify dysfunctional tumor-reactive CD8^+^ T cells. A major population of CD8^+^ TILs co-expressing 4-1BB and LAG-3 was observed in the “progressor” tumor models B16 and C1498 but not the spontaneously rejected “regressor” MC57.SIY model. Kinetic analysis showed the appearance and persistence of this population over time only in the tumor. Upon *ex vivo* stimulation the 4-1BB^+^LAG-3^+^ as well as CD8+Egr2GFP^+^ TILs exhibited a significant reduction in IL-2 production compared to their negative counterpart indicating TIL dysfunction. TCR repertoire analysis showed CDR3β skewing in the 4-1BB^+^LAG-3^+^ compartment, indicating oligoclonality. In addition, CD8^+^ TIL specific for the model antigen SIY expressed LAG-3 and 4-1BB. Further, 2C T cells transferred into a tumor bearing host upregulated LAG-3 and 4-1BB within 3 days after entering the tumor. These data confirmed that the vast majority of tumor-reactive CD8^+^ TILs express 4-1BB and LAG-3. Although the 4-1BB^+^LAG-3^+^ TIL population lacked the ability to produce IL-2, high levels of IFN-g, GzmB, Perforin, IL-10 as well as the Treg-recruiting chemokines CCL1 and CCL22 were produced by this population. These data argue that this population may exert negative immunomodulatory functions and not be completely inert. Therapeutic targeting of this population by agonistic 4-1BB and blocking LAG-3 mAb combination resulted in increased tumor control and an overall shift towards short term effector KLRG1^hi^IL7R^lo^ phenotype in CD8^+^ TILs as well as decreased expression of several inhibitory receptors including PD-1 and 2B4. Interestingly, *ex vivo* stimulation of CD8^+^ TILs after treatment resulted in restoration of the ability to produce IL-2. Our results suggest that the co-expression of LAG-3 and 4-1BB identifies a critical subpopulation of dysfunctional tumor antigen-specific CD8^+^ TILs and may contribute to an immune suppressive tumor microenvironment. Inhibiting LAG-3 while agonizing 4-1BB resulted in an increased anti-tumor response and a significant change in phenotype and function in the CD8^+^ TIL compartment.

